# Supplementary material for: Transcriptomic analyses implicate neuronal plasticity and chloride homeostasis in ivermectin resistance and response to treatment in a parasitic nematode
Source: PLoS Pathog. 2022 Jun 13;18(6):e1010545. doi: 10.1371/journal.ppat.1010545 (PMC9232149; doi:10.1371/journal.ppat.1010545)
Supplement: S1 Fig — A. The Haemonchus contortus MHco3 isolate is fully drug susceptible and the MHco18 isolate is multi-drug resistant; these are the two parental populations used to generate the genetic cross, of which the parent and F2 adult populations are used in this study. One donor sheep was infected per population. Nomenclature, drug treatment, expected resistance phenotype(s) and adult worm samples used in our analyses are described. B. Pairwise comparisons of differential gene expression included in each analysis. For each analysis, genes were filtered for significant differential expressed (adjusted P<0.01) in all pairwise comparisons marked ✓ and for non-significant differential expression (adjusted P>0.01) in all pairwise comparisons marked ✕. Pairwise comparisons marked—were not included in the analysis. Genes with significant differential expression were filtered to retain only those where the direction of log fold change (>0 or <0) was the same in all pairwise comparisons i.e. for inclusion, a gene could not be upregulated in a resistant population in one pairwise comparison and downregulated in a resistant population in another. (PDF) [file ppat.1010545.s001.pdf]

A

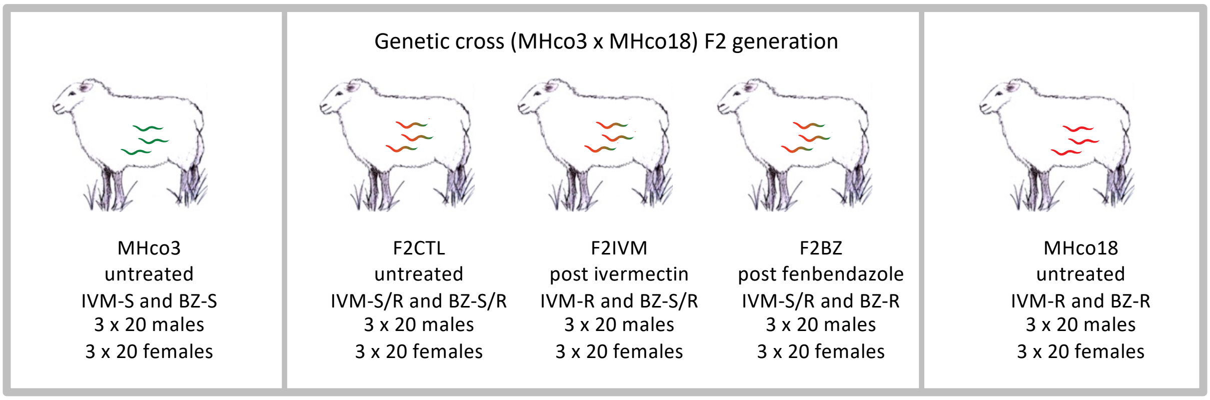

B

Analysis \ Pairwise comparison	MHco18 v MHco3 males	MHco18 v MHco3 females	F2IVM v MHco3 males	F2IVM v MHco3 females	F2IVM v F2CTL males	F2BZ v MHco3 males	F2BZ v MHco3 females	F2BZ v F2CTL males	MHco4 v MHco3 females	MHco10 v MHco3 females
Transcriptomic differences associated with ivermectin resistance	✓	✓	✓	✓	✓	-	-	-	-	-
Differences in gene expression in ivermectin resistant male worms	✓	✕	✓	✕	✓	-	-	-	-	-
Differences in gene expression in ivermectin resistant female worms	✕	✓	✕	✓	✕	-	-	-	✓	✓
Transcriptomic response to ivermectin exposure	✕	✕	✓	✓	✓	-	-	-	-	-
Similarities in gene expression with benzimidazole selected populations	✓	✓	✓	✓	✓	✓	✓	✓	-	-
Transcriptomic differences associated with benzimidazole resistance	✓	✓	-	-	-	✓	✓	✓	-	-
