# Supplementary figures and images for: Transcriptomic analyses implicate neuronal plasticity and chloride homeostasis in ivermectin resistance and response to treatment in a parasitic nematode
Source: PLoS Pathog. 2022 Jun 13;18(6):e1010545. doi: 10.1371/journal.ppat.1010545 (PMC9232149; doi:10.1371/journal.ppat.1010545)

A

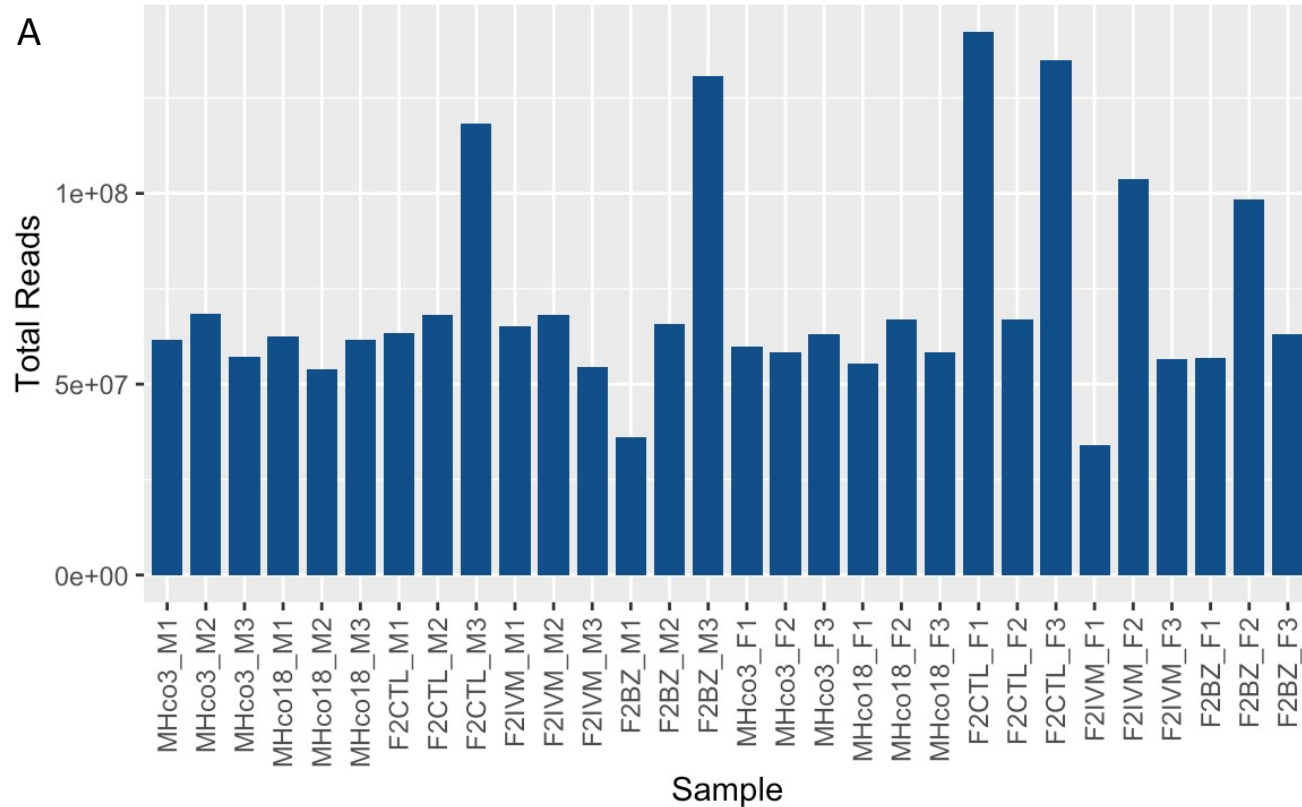

B

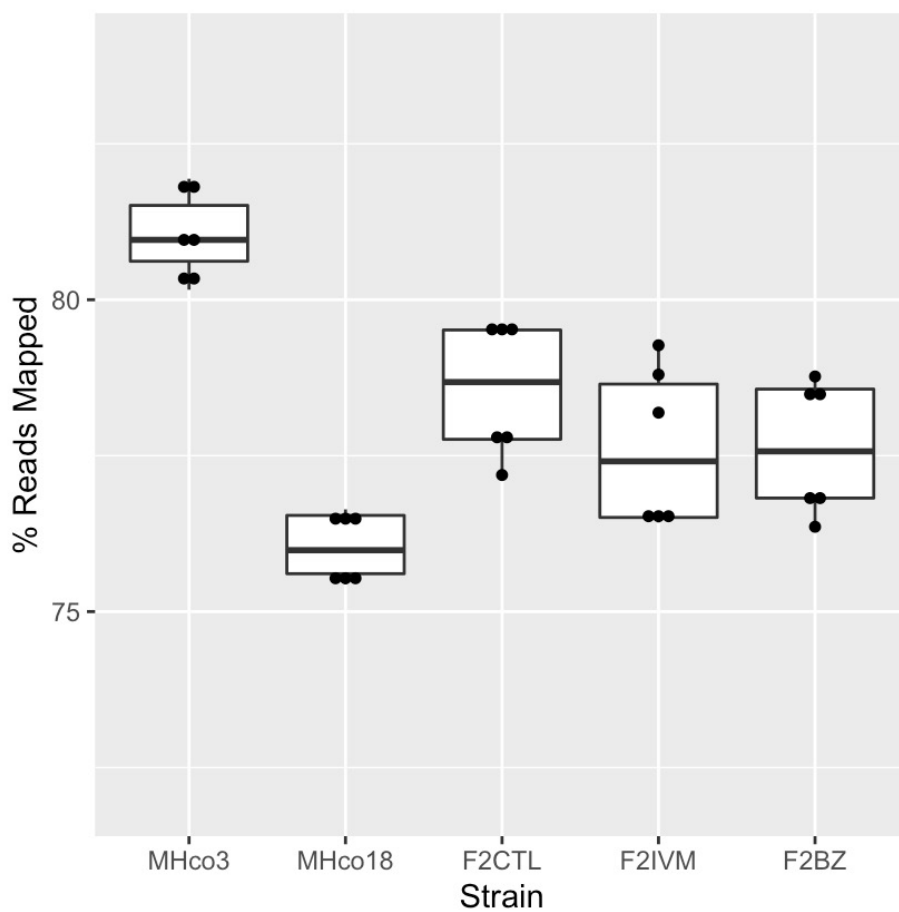

Supplement: S2 Fig — A. Total reads sequenced for each sample. B. Percentage of reads mapped to MHco3 reference genome for each strain. (PDF) [file ppat.1010545.s002.pdf]

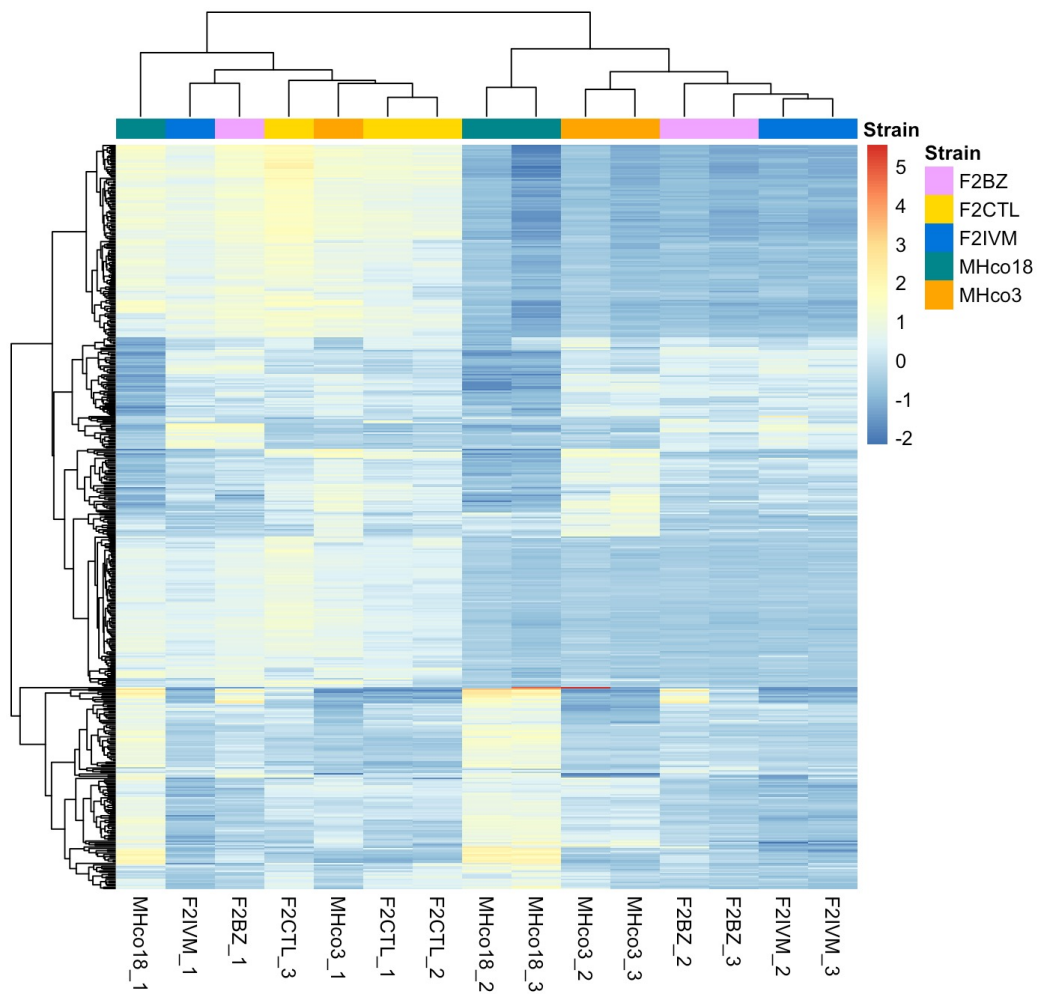

Supplement: S3 Fig — Two clear clusters appear: the F2CTL samples plus sample 1 of every other group (left branch) and all other samples (right branch). (PDF) [file ppat.1010545.s003.pdf]

A

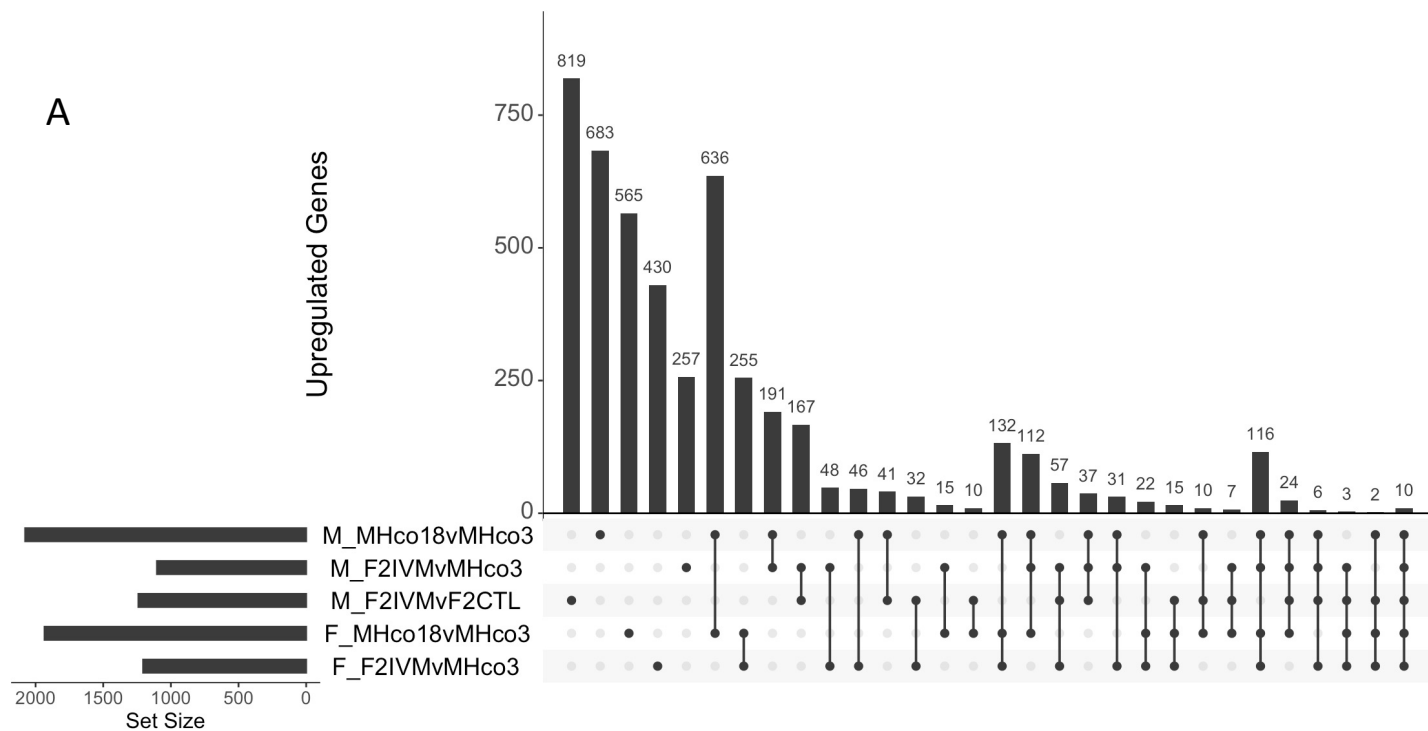

B

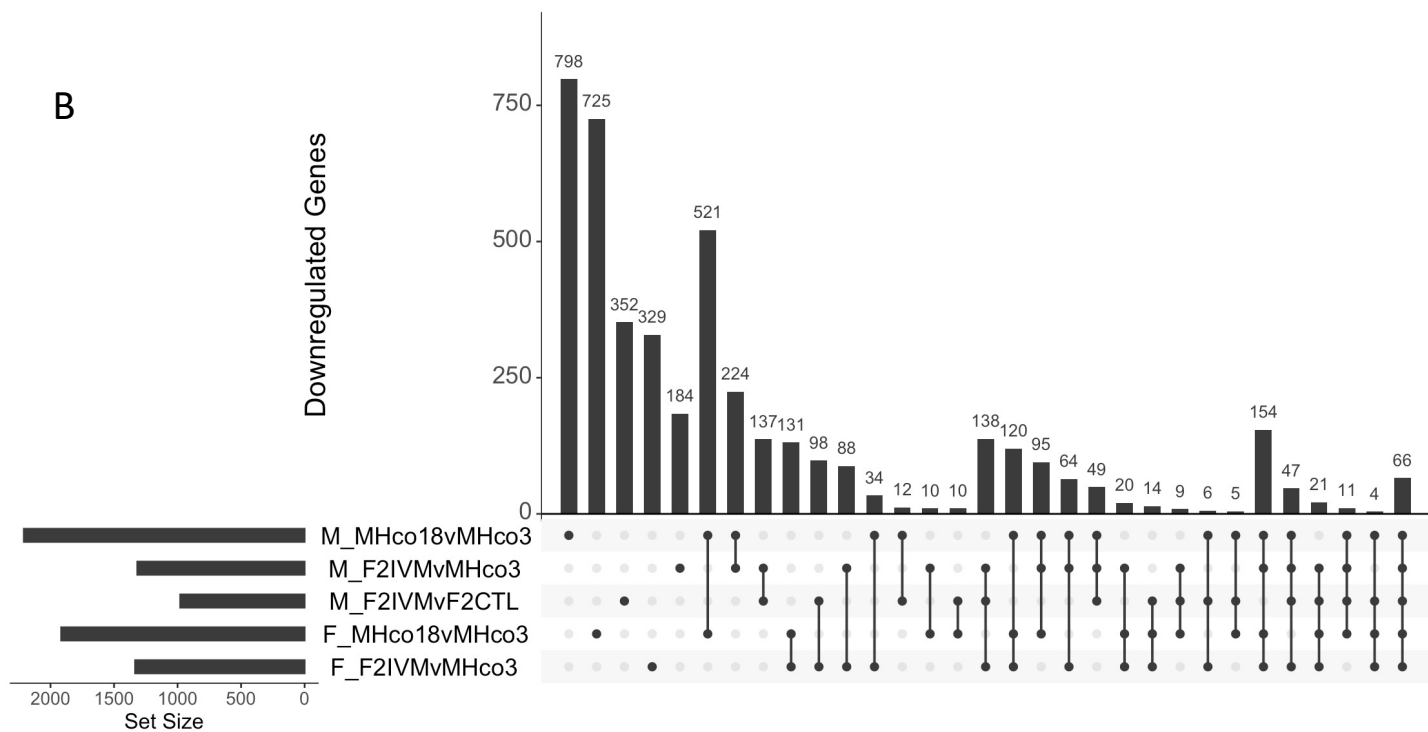

Supplement: S4 Fig — Upset plots showing the number of shared upregulated (A) and downregulated (B) genes in different pairwise comparisons for male (M) and female (F) samples. (PDF) [file ppat.1010545.s004.pdf]

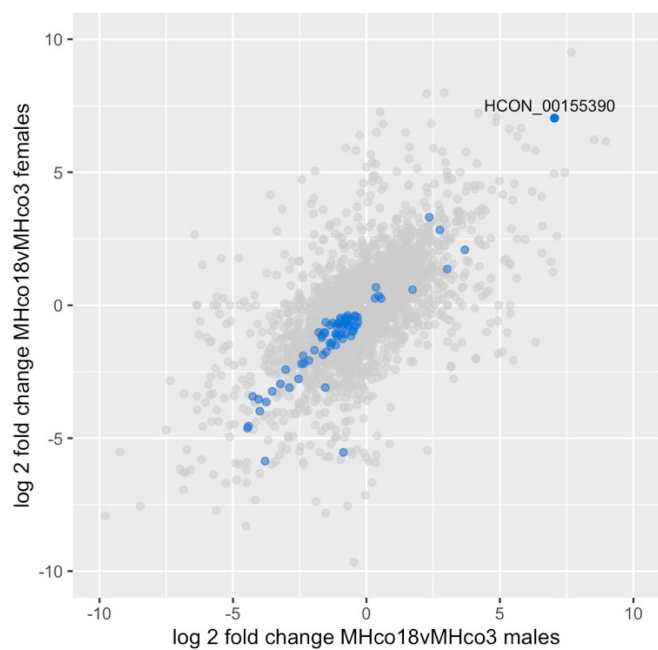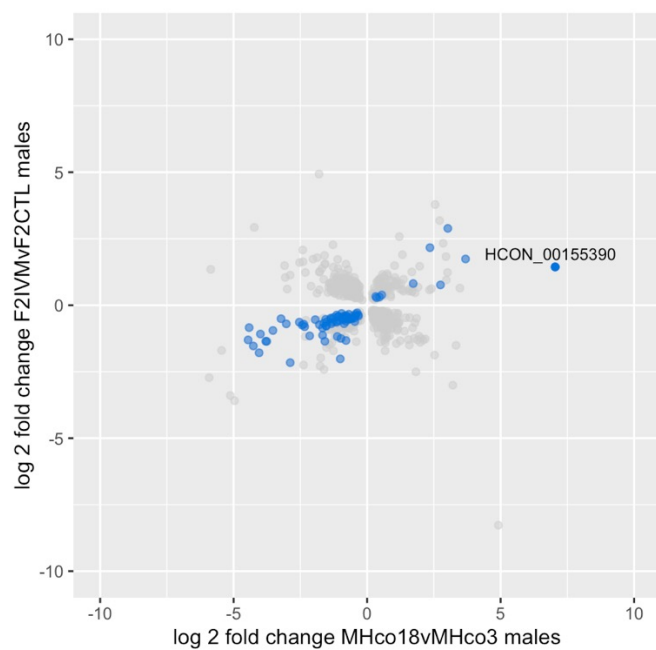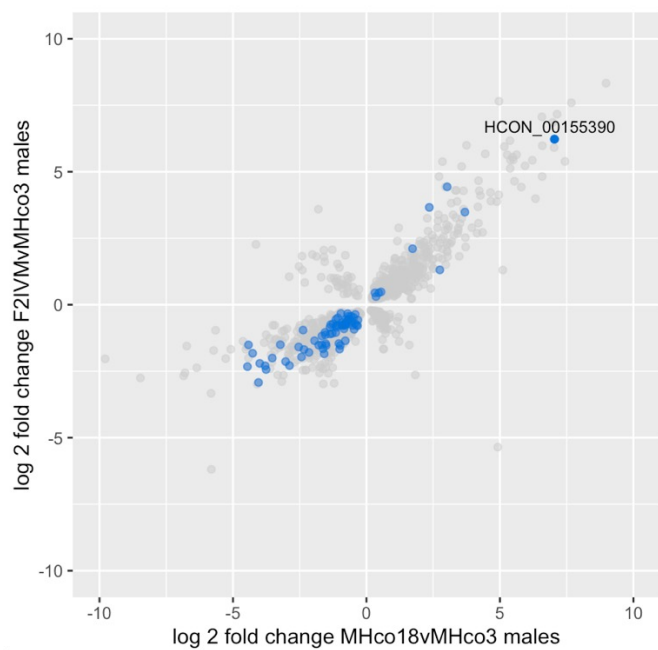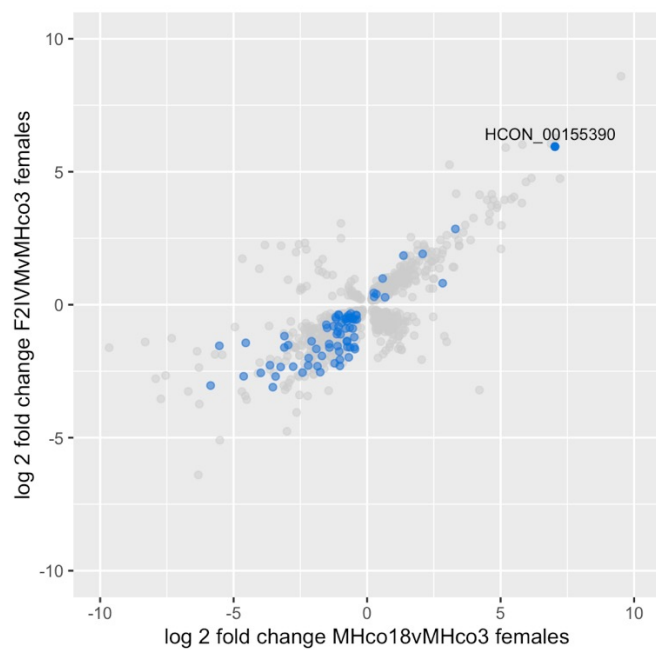

Supplement: S5 Fig — Grey points represent genes that are differentially expressed in the pairwise comparison on each axis. If blue they are differentially expressed in every resistant and susceptible pairwise comparison (MHco18vMHco3, F2IVMvF2CTL (males only) and F2IVMvMHco3). HCON_00155390 is the H. contortus homologue of C. elegans cky-1. (PDF) [file ppat.1010545.s005.pdf]

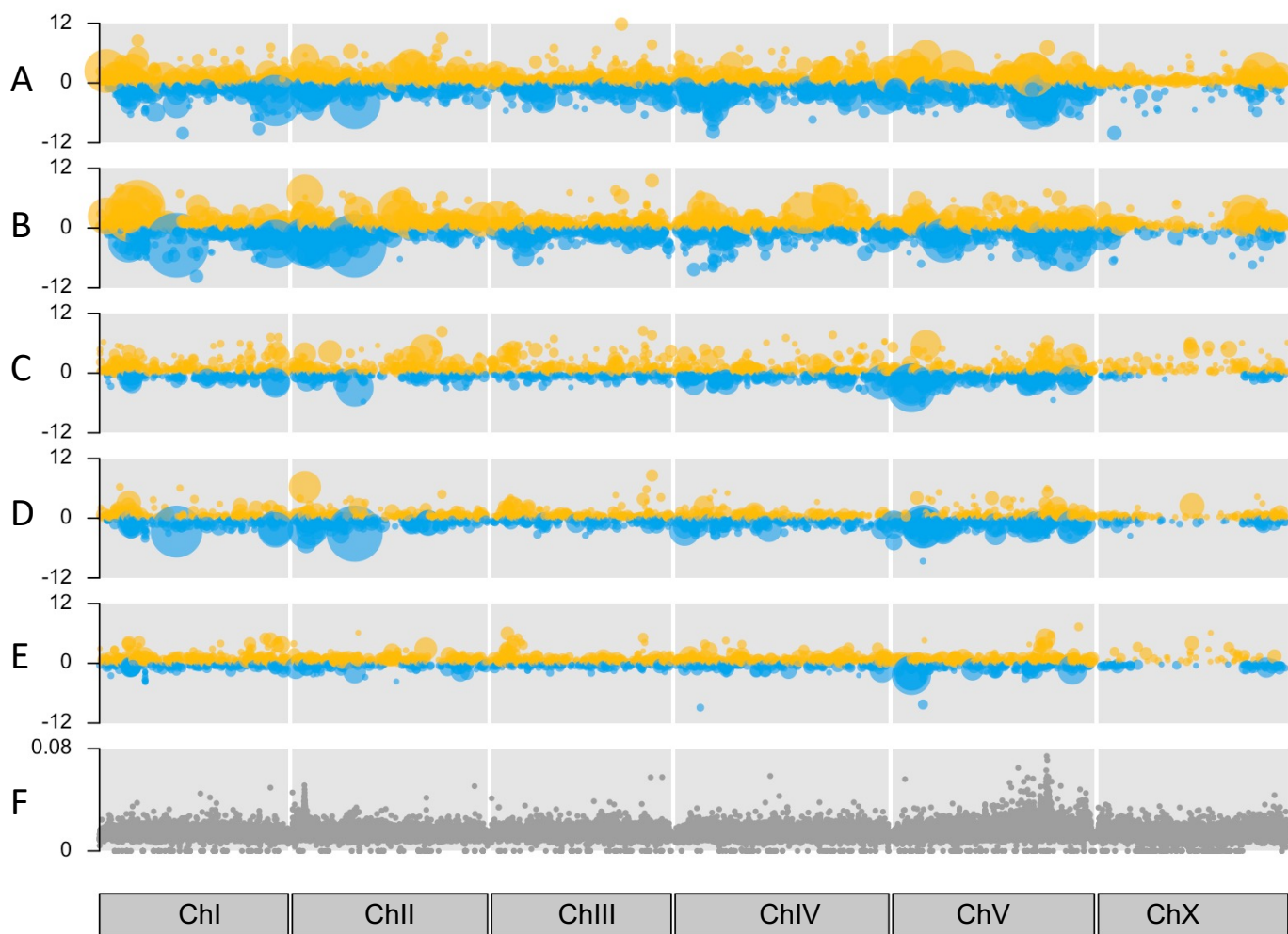

Supplement: S6 Fig — Genome-wide karyoplots showing genomic loci of genes with significant upregulation (yellow) or downregulation (blue). Point size corresponds to significance. A. MHco18 vs MHco3 males, B. MHco18 vs MHco3 females, C. F2IVM vs MHco3 males, D. F2IVM vs MHco3 females and E. F2IVM vs F2CTL males. Panel F shows genetic differentiation (FST) between the F3 generation of the genetic cross pre- and post- ivermectin selection [18]. (PDF) [file ppat.1010545.s006.pdf]

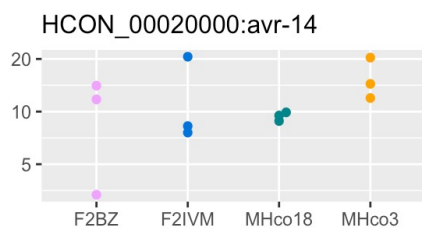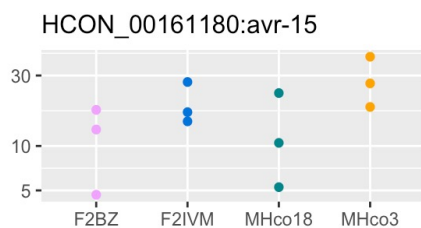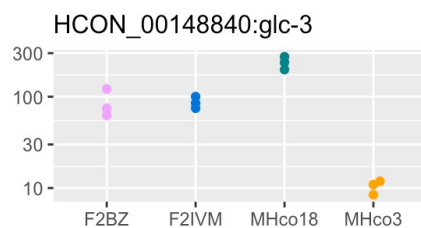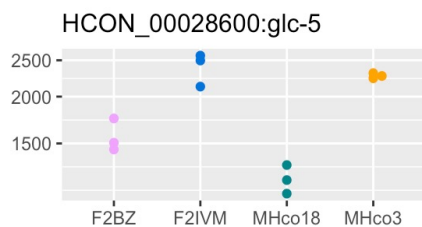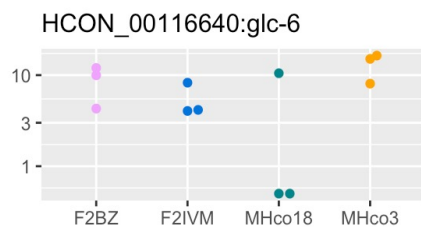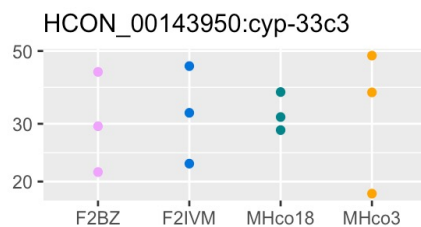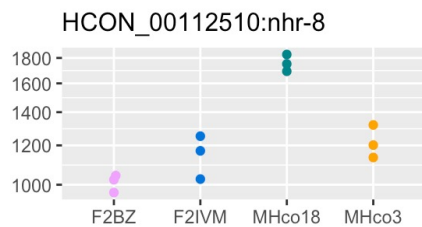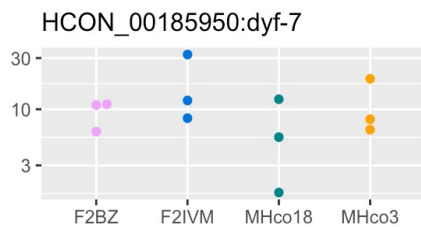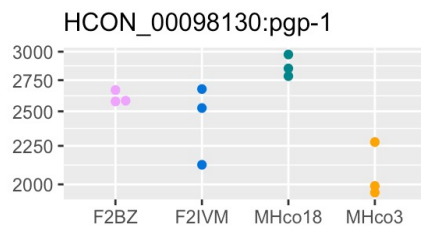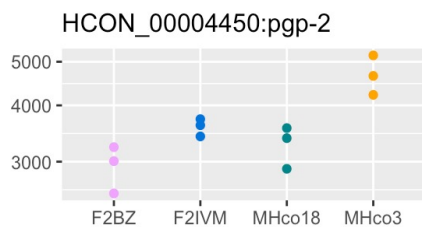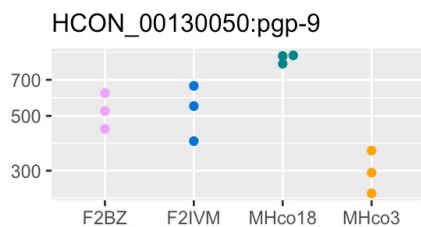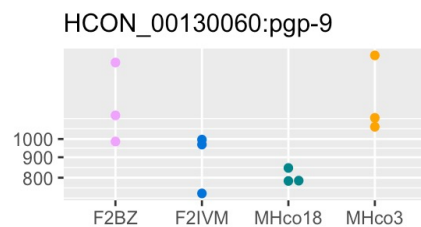

Supplement: S8 Fig — (PDF) [file ppat.1010545.s008.pdf]

Male only

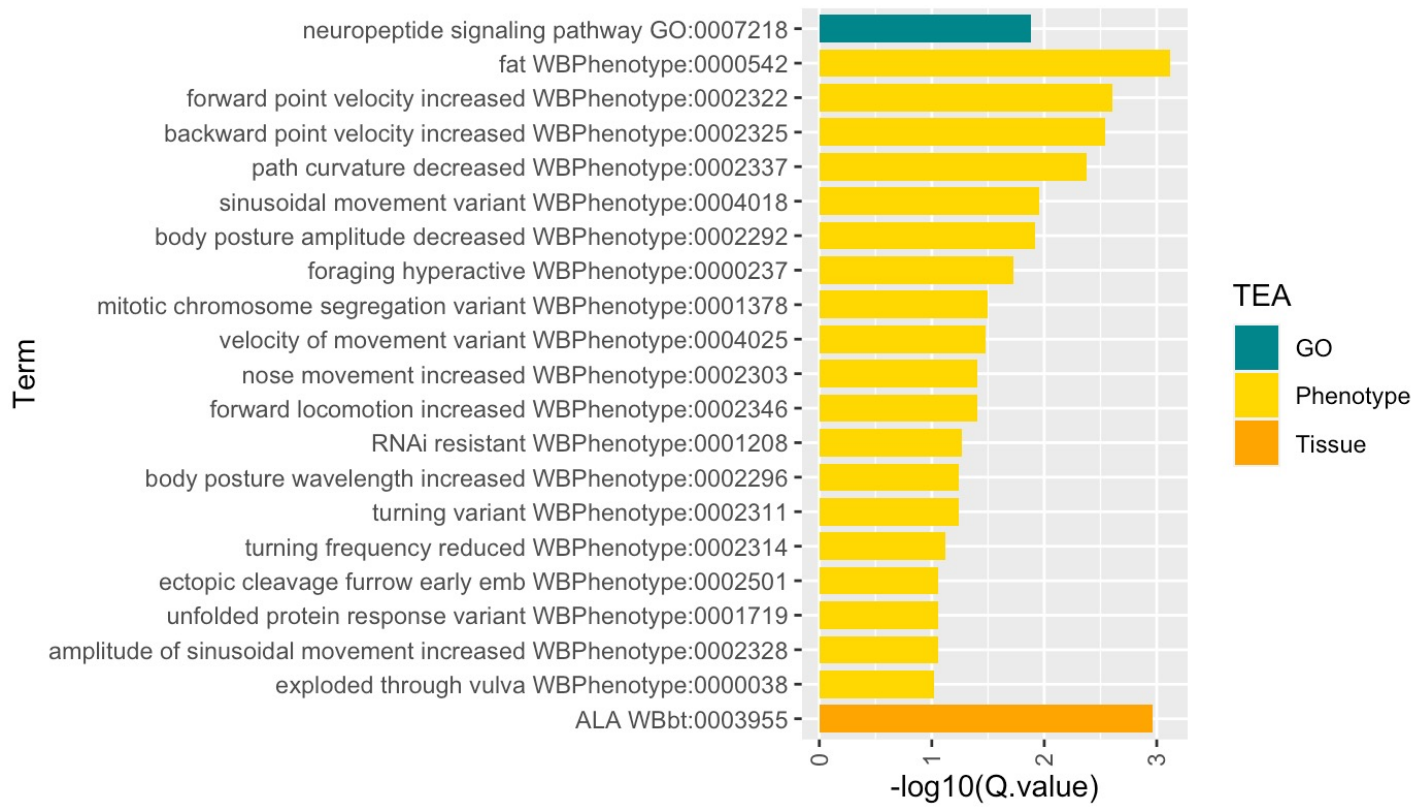

Response to ivermectin treatment

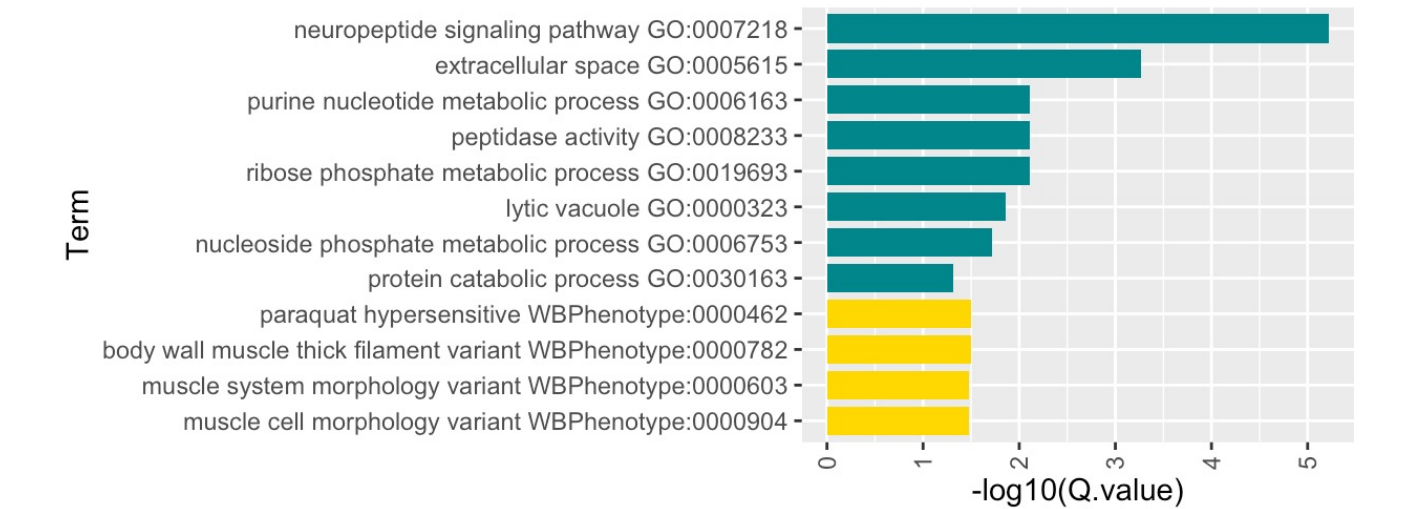

Supplement: S9 Fig — (PDF) [file ppat.1010545.s009.pdf]

A

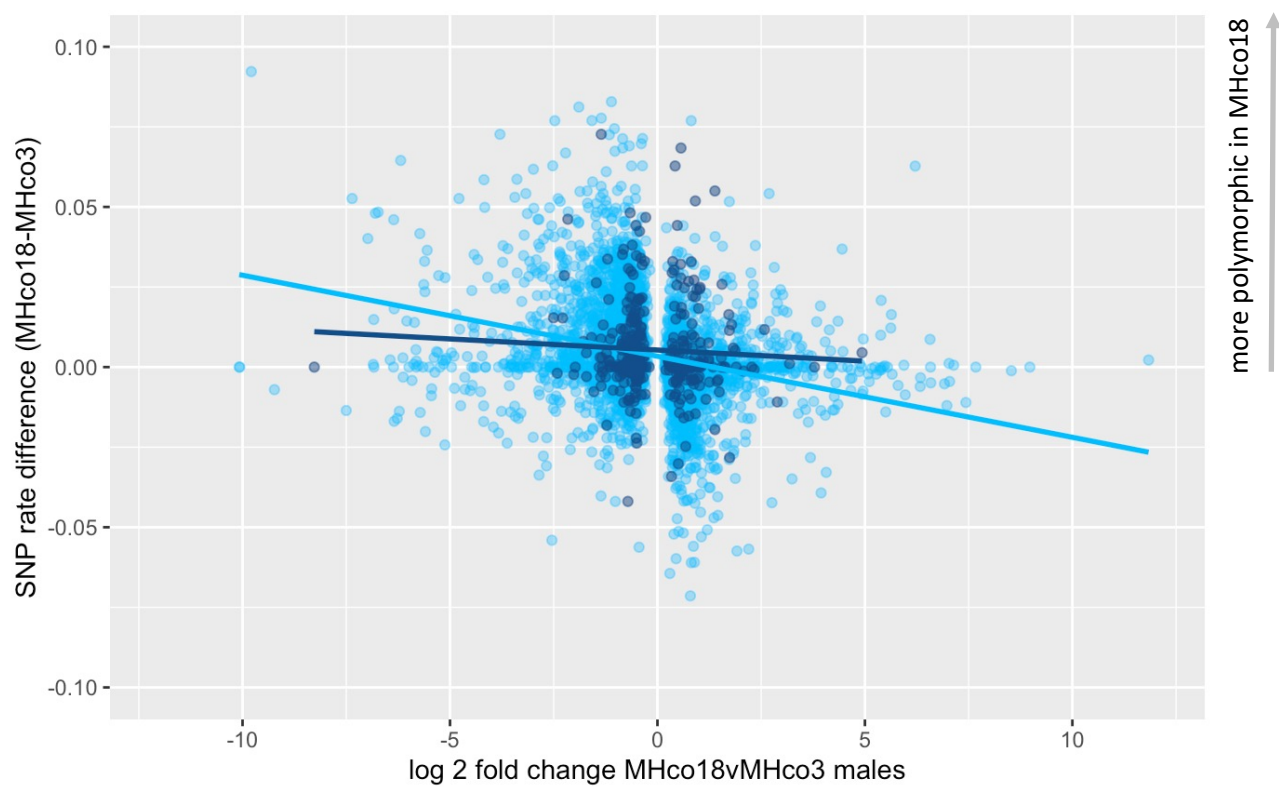

B

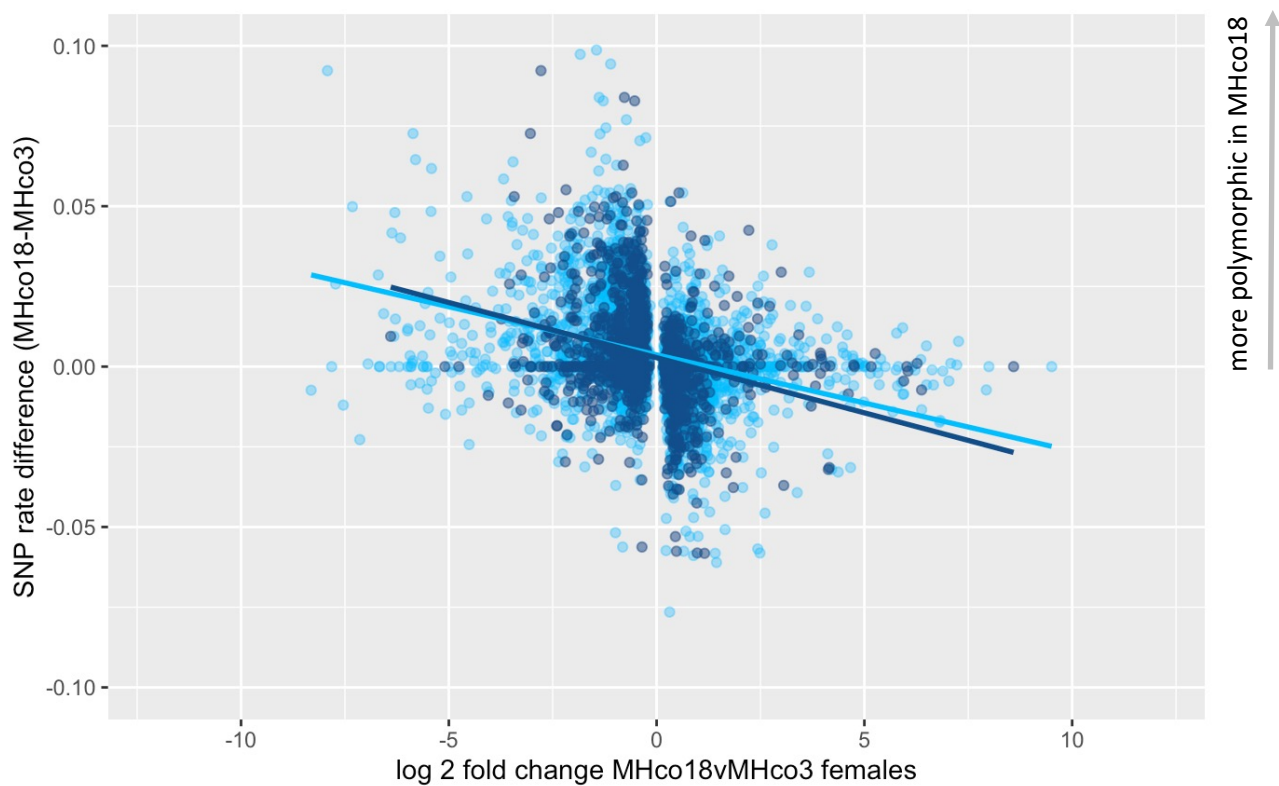

Supplement: S10 Fig — Scatter plots showing SNP rate versus differential expression in males (A) and females (B). Points represent genes that are differentially expressed in the parental isolates: light blue if differentially expressed in MHco18 vs MHco3 only, dark blue if also differentially expressed in F2IVM vs F2CTL and F2IVM vs MHco3 (males) or F2IVM vs MHco3 (females). (PDF) [file ppat.1010545.s010.pdf]
