# Supplementary material for: Transcriptomic analyses implicate neuronal plasticity and chloride homeostasis in ivermectin resistance and response to treatment in a parasitic nematode
Source: PLoS Pathog. 2022 Jun 13;18(6):e1010545. doi: 10.1371/journal.ppat.1010545 (PMC9232149; doi:10.1371/journal.ppat.1010545)
Supplement: S7 Fig — (PDF) [file ppat.1010545.s007.pdf]

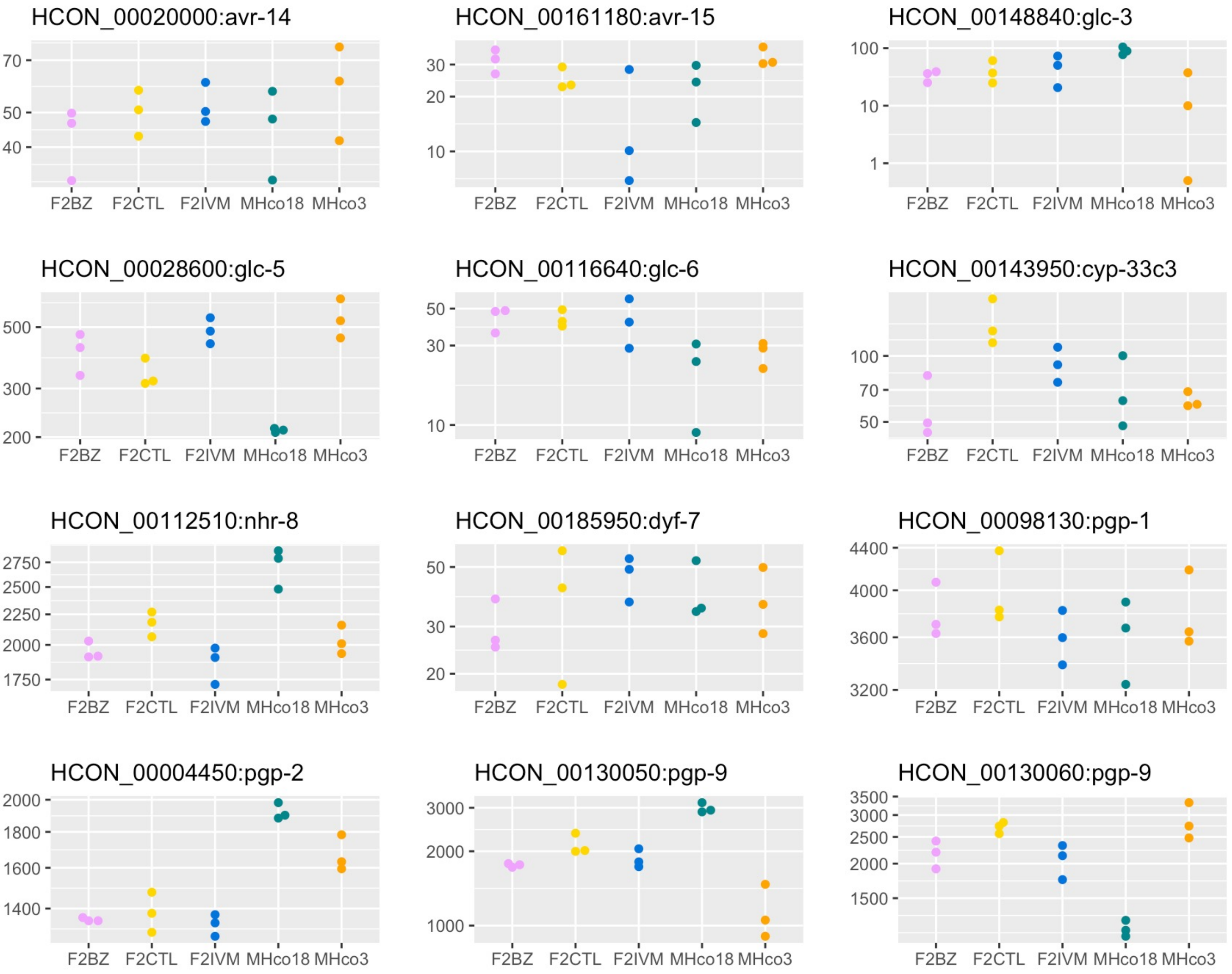
HCON_00020000:avr-14
70
50
40
HCON_00161180:avr-15
30
20
10
HCON_00148840:glc-3
100
10
1
HCON_00028600:glc-5
500
300
200
HCON_00116640:glc-6
50
30
10
HCON_00143950:cyp-33c3
100
70
50
HCON_00112510:nhr-8
2750
2500
2250
2000
1750
HCON_00185950:dyf-7
50
30
20
HCON_00098130:pgp-1
4400
4000
3600
3200
HCON_00004450:pgp-2
2000
1800
1600
1400
HCON_00130050:pgp-9
3000
2000
1000
HCON_00130060:pgp-9
3500
3000
2500
2000
1500
F2BZ
F2CTL
F2IVM
MHco18
MHco3
